# Sleep deficiency and motor vehicle crash risk in the general population: a prospective cohort study

**DOI:** 10.1186/s12916-018-1025-7

**Published:** 2018-03-20

**Authors:** Daniel J. Gottlieb, Jeffrey M. Ellenbogen, Matt T. Bianchi, Charles A. Czeisler

**Affiliations:** 1 Division of Sleep and Circadian Disorders, Departments of Medicine and Neurology, Brigham & Women’s Hospital, 221 Longwood Ave, BLI 225E, Boston, MA 02115 USA; 2 VA Boston Healthcare System, Boston, MA USA; 3 Division of Sleep Medicine, Harvard Medical School, Boston, MA USA; 4 Department of Neurology, Massachusetts General Hospital, Boston, MA USA

**Keywords:** Motor vehicle crash, Motor vehicle accident, Sleep deficiency, Sleep apnea, Sleep duration, Sleep deprivation, Epidemiology

## Abstract

**Background:**

Insufficient sleep duration and obstructive sleep apnea, two common causes of sleep deficiency in adults, can result in excessive sleepiness, a well-recognized cause of motor vehicle crashes, although their contribution to crash risk in the general population remains uncertain. The objective of this study was to evaluate the relation of sleep apnea, sleep duration, and excessive sleepiness to crash risk in a community-dwelling population.

**Methods:**

This was a prospective observational cohort study nested within the Sleep Heart Health Study, a community-based study of the health consequences of sleep apnea. The participants were 1745 men and 1456 women aged 40–89 years. Sleep apnea was measured by home polysomnography and questionnaires were used to assess usual sleep duration and daytime sleepiness. A follow-up questionnaire 2 years after baseline ascertained driving habits and motor vehicle crash history. Logistic regression analysis was used to examine the relation of sleep apnea and sleep duration at baseline to the occurrence of motor vehicle crashes during the year preceding the follow-up visit, adjusting for relevant covariates. The population-attributable fraction of motor vehicle crashes was estimated from the sample proportion of motor vehicle crashes and the adjusted odds ratios for motor vehicle crash within each exposure category.

**Results:**

Among 3201 evaluable participants, 222 (6.9%) reported at least one motor vehicle crash during the prior year. A higher apnea-hypopnea index (*p* < 0.01), fewer hours of sleep (*p* = 0.04), and self-reported excessive sleepiness (*p* < 0.01) were each significantly associated with crash risk. Severe sleep apnea was associated with a 123% increased crash risk, compared to no sleep apnea. Sleeping 6 hours per night was associated with a 33% increased crash risk, compared to sleeping 7 or 8 hours per night. These associations were present even in those who did not report excessive sleepiness. The population-attributable fraction of motor vehicle crashes was 10% due to sleep apnea and 9% due to sleep duration less than 7 hours.

**Conclusions:**

Sleep deficiency due to either sleep apnea or insufficient sleep duration is strongly associated with motor vehicle crashes in the general population, independent of self-reported excessive sleepiness.

## Background

Sleep deficiency, defined as insufficient “quantity or quality of sleep” for optimal health and performance, may result from inadequate total sleep duration or from fragmentation of sleep [1]. The potential health impact of insufficient sleep duration was first reported in the 1960s, when a general population survey of over one million adults found that individuals who reported sleeping fewer than 7 hours per night had increased mortality compared to those sleeping 7 to 8 hours per night [2, 3]. Recent data indicate that usual sleep duration has decreased substantially since that time, with an estimated 25–30% of U.S. adults now sleeping 6 or fewer hours per night, a sleep duration associated with obesity, hypertension, glucose intolerance, diabetes mellitus, coronary heart disease, and death [4]. Restricted sleep is also an important cause of excessive sleepiness, a well-recognized cause of motor vehicle crashes [5–7]. Data from the U.S. Centers for Disease Control indicate that habitual sleep duration of 6 or fewer hours per night is associated with a 2.6-fold higher risk of reporting having fallen asleep while driving, compared to those sleeping 7–9 hours per night, although motor vehicle crashes were not assessed [8]. Although there are individual differences in impairment from sleep deprivation [9], the perception of impairment quickly plateaus in individuals whose sleep duration is chronically restricted, despite continued declines in objective measures of performance [10, 11], suggesting that individuals may be unaware of their degree of impairment from sleep deficiency.

Obstructive sleep apnea is a common chronic disorder in adults and it is associated with excessive sleepiness [12]. The prevalence of sleep apnea has increased with the increase in overweight and obesity. Estimates based on current U.S. population demographics indicate that 17% of women and 34% of men aged 30–70 have sleep apnea [13], with 5% and 14%, respectively, having both sleep apnea and self-reported excessive sleepiness. Like insufficient sleep duration, sleep apnea is associated with hypertension, diabetes mellitus, cardiovascular disease, and mortality [4, 14–17]. Sleep apnea is also associated with motor vehicle crash risk [18]. A recent meta-analysis concluded that patients with obstructive sleep apnea have a crash risk approximately 2.5 times that of individuals without sleep apnea, but the data were equivocal whether the excess crash risk was explained solely on the basis of excessive sleepiness [19]. While a diagnosis of obstructive sleep apnea can be made in the absence of excessive sleepiness [20], the importance of asymptomatic sleep apnea has been questioned. A recent U.S. Preventive Services Task Force recommendation concluded that “the current evidence is insufficient to assess the balance of benefits and harms of screening for obstructive sleep apnea in asymptomatic adults” [21].

As self-perceived excessive sleepiness is absent in more than half of individuals with sleep apnea in the general population, even among those with severe sleep apnea [13, 22], it remains an important unresolved question whether these individuals are at increased motor vehicle crash risk. Similarly, it has been argued that some individuals are naturally short sleepers and that failure to obtain an optimal duration of sleep is inconsequential in the absence of subjective sleepiness. In the present analysis, we ask whether individuals with sleep deficiency, due either to short usual sleep duration or obstructive sleep apnea, are at increased risk of motor vehicle crashes, independent of self-reported daytime sleepiness.

## Methods

### Study design

The Sleep Heart Health Study is a community-based prospective cohort study of the cardiovascular consequences of sleep apnea. Briefly, 6441 men and women aged 40 years and older were recruited from existing population-based studies, as previously described [23, 24]. Therapy for sleep apnea or use of nocturnal supplemental oxygen were exclusion criteria. At baseline, participants completed sleep habits and general health questionnaires and underwent overnight polysomnography. Approximately 2 years later, the participants again completed sleep habits and general health questionnaires, including questions related to driving habits and history of motor vehicle crashes. The aim of the present investigation is to determine whether sleep apnea or short sleep duration are associated with increased crash risk in the general population, and whether self-reported sleepiness identifies those at increased crash risk. The protocol was approved by the Institutional Review Board of each participating center and signed informed consent was provided by each participant. The full Sleep Heart Health Study protocol, including the sleep habits, sleepiness and driving history questionnaires, are available at http://jhuccs1.us/shhs/details/studydoc.htm.

### Study sample

**Fig. 1:**
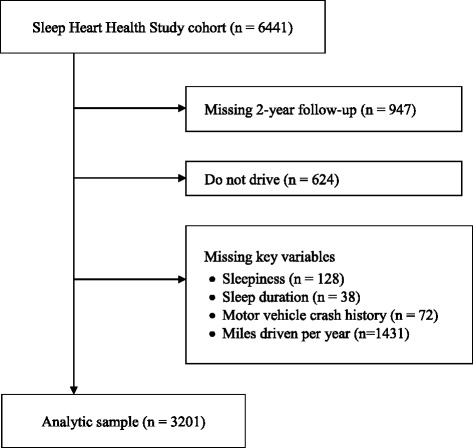
Ascertainment of the study sample

The composition of the study sample is shown in Fig. 1. Of the 6441 Sleep Heart Health Study participants, 2-year follow-up questionnaire data were available for 5494 (85.3%). Of these, 624 non-drivers were excluded, as were 238 with missing data on sleepiness, sleep duration, or motor vehicle accidents. Of the remaining 4632 individuals, 3201 reported the average number of miles driven per year. Given the strong association between miles driven and motor vehicle crash risk, these 3201 individuals are the primary sample analyzed for this report. The included sample was slightly younger (62.3 vs. 63.5 years, *p* < 0.001) and had a higher percentage of men (54.5% vs. 39.9%, *p* < 0.001) than the 3240 participants who were excluded for any reason. Their reported usual sleep duration was slightly longer (7.1 vs. 7.0 hours/night, *p* < 0.01), and they were similar to excluded participants in mean apnea-hypopnea index (AHI, 8.7 vs. 9.0, *p* = 0.36) and Epworth Sleepiness Scale scores (7.9 vs 7.7, *p* = 0.34). Of the 3201 included participants, 2340 reported the usual number of hours driven per day, while 1295 participants were excluded because they did not report the number of miles driven per year but did report the usual number of hours driven per day. There was no significant difference between included and excluded participants in reported usual hours of driving per day (1.83 versus 1.86 hours per day, *p* = 0.54).

### Sleep measures

Participants underwent a single night of in-home polysomnography at baseline, as previously described [25, 26]. AHI was defined as the average number of apneas plus hypopneas per hour of sleep, where both apneas and hypopneas required an associated 4% or greater oxygen desaturation. Usual sleep duration was defined as the response to the question, “How many hours of sleep do you usually get at night (or your main sleep period) on weekdays or workdays?” Responses were integer values. The stability of this measure over time in this cohort has been previously reported [27]. Sleepiness was assessed using the Epworth Sleepiness Scale, an eight-item self-completion questionnaire that asks the participant to rate his/her likelihood of falling asleep in a variety of commonly encountered situations [28]. Excessive sleepiness was defined as a score ≥11 on this scale.

### Driving history

As part of a general follow-up questionnaire completed approximately 2 years after baseline, participants were asked, “Do you drive?” Those answering affirmatively were further asked about driving frequency, average time spent driving per day or miles driven per year, and the question: “How many accidents have you had in the last year while you were the driver?”

### Statistical analysis

All statistical analyses were performed using SAS version 9.2 (SAS Institute, Inc., Cary, NC). Descriptive statistics are presented comparing participants with and without motor vehicle crash, with independent sample *t* tests used to compare means of continuous variables and Fisher’s exact test to compare proportions. Logistic regression analysis was used to examine the relation of sleep apnea and sleep duration at baseline to the occurrence of at least one motor vehicle crash during the year preceding the follow-up visit, adjusting for relevant covariates. The primary models adjusted for age, sex, and miles driven. The Epworth Sleepiness Scale score was added to the models to assess mediation by self-reported sleepiness of the effects of sleep apnea and usual sleep duration, and models restricted to those without excessive sleepiness were run to determine whether excess crash risk was present in those without self-reported sleepiness. Models that included both sleep apnea and sleep duration measures, with an appropriate interaction term, were used to assess a possible synergy between sleep apnea and sleep deprivation in relation to crash risk. Sensitivity analyses including those individuals missing data on miles driven, adjusted only for age and sex, were conducted to determine whether their exclusion might have biased the study results. Additional models adjusted for alcohol consumption, smoking history, body mass index, sleeping pill use, and caffeine consumption; explored the independent effect of habitual snoring on crash risk; explored possible sex differences in the effect of sleep deficiency on crash risk; and explored differences in the effect of sleep deficiency between older and younger participants. The population-attributable fraction of motor vehicle crashes was estimated from the sample proportion of motor vehicle crashes in each exposure category and the adjusted odds ratios for motor vehicle crash in those categories, as described by Miettinen [29].

## Results

**Fig. 2:**
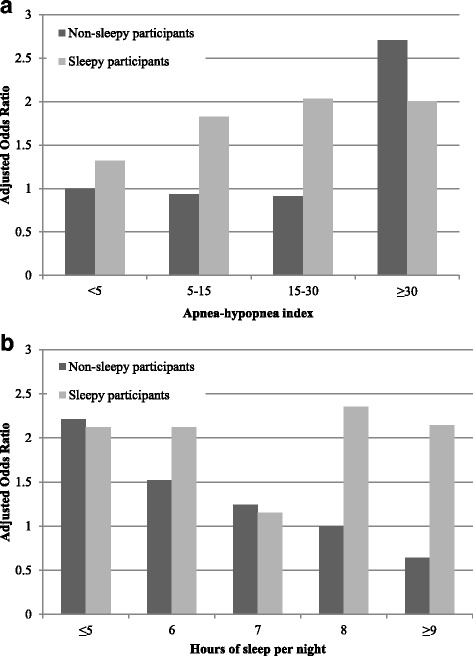
The relation of sleep apnea (**a**) and reported usual sleep duration (**b**) to motor vehicle crash risk, adjusted for age, sex, and miles driven. Data are shown separately for non-sleepy (black, *n* = 2402) and sleepy (gray, *n* = 799) participants, where sleepiness is defined as a score ≥ 11 on the Epworth Sleepiness Scale. The referent group for **a** is non-sleepy participants with apnea-hypopnea index < 5. The referent group for **b** is non-sleepy participants reporting a usual sleep duration of 8 hours per night

At least one motor vehicle crash during the year prior to follow-up evaluation was reported by 6.9% of the 3201 participants. Motor vehicle crashes were somewhat more common in men than in women and were significantly associated with number of miles driven per year, AHI, sleep duration, and self-reported sleepiness (Table 1). Adjusted for age, sex, and miles driven, the odds ratio for any motor vehicle crash increased by 15% for every 10-unit increase in AHI in the overall population and by 17% for every 10-unit increase in AHI in participants who did not report excessive sleepiness (Table 2). Although non-linear models were not a significantly better fit to the data, the excess risk in self-reported non-sleepy participants was apparent only in those with severe sleep apnea (Fig. 2a). Compared to those without sleep apnea (AHI < 5, *n* = 1730), those with mild (AHI 5 to < 15, *n* = 913), moderate (AHI 15 to < 30, *n* = 364), and severe (AHI ≥30, *n* = 194) sleep apnea had adjusted odds ratios (aOR) for any motor vehicle crash that were respectively 7%, 13% and 123% higher. The population-attributable fraction of motor vehicle crashes due to sleep apnea was 10%. Habitual snoring was not significantly associated with crash risk in models that included measures of sleep apnea.

**Table 1:** Baseline characteristics of the study sample

Characteristic	No MVC (*n* = 2979)^*^	MVC (*n* = 222)^†^	*P* value
Age – years	62.3 (10.1)	61.5 (10.8)	0.22
Male sex – no. (%)	1610 (54.0)	135 (60.8)	0.06
Body mass index – kg/m^2^	28.2 (4.9)	28.6 (5.6)	0.31
Comorbid illness – no. (%)			
Diabetes mellitus	192 (6.7)	15 (7.3)	0.66
Hypertension	1174 (39.4)	83 (37.4)	0.57
Myocardial infarction	186 (6.3)	11 (5.0)	0.34
Stroke	73 (2.5)	5 (2.3)	0.65
Smoking status – no. (%)			0.96
Current	297 (10.0)	21 (9.5)	
Former	1367 (46.1)	103 (46.4)	
Alcohol consumption – drinks per week	1 (0, 4)	0 (0, 3)	0.88
Caffeine consumption – drinks per day	2 (1, 4)	2 (1, 4)	0.76
Sleeping pill use – no. (%)	1989 (6.7)	19 (8.6)	0.27
Epworth Sleepiness Scale score	7.7 (4.3)	8.6 (4.7)	< 0.01
Apnea-hypopnea index – events per hour	4.3 (1.3, 10.9)	5.5 (1.3, 13.7)	< 0.01
Usual sleep duration – hours per night	7.1 (1.1)	6.9 (1.1)	0.02
Miles driven per year – thousands	9.0 (4.0, 14.0)	11.5 (7.0, 15.0)	< 0.001

Values are number (%) or mean (standard deviation), with the exception of alcohol and caffeine consumption, apnea-hypopnea index, and miles driven, which are median (first quartile, third quartile)

*MVC* motor vehicle crash

^*^Missing data in the sample without MVC: body mass index (*n* = 4), diabetes (*n* = 91), myocardial infarction (*n* = 26), stroke (*n* = 26), smoking status (*n* = 16), alcohol consumption (*n* = 206), sleeping pill use (*n* = 6)

^†^Missing data in the sample with MVC: body mass index (*n* = 3), diabetes (*n* = 16), myocardial infarction (*n* = 1), stroke (*n* = 1), alcohol consumption (*n* = 24), sleeping pill use (*n* = 1)

**Table 2:** Relation of sleep apnea, sleep duration, and self-reported sleepiness to motor vehicle crash risk

Characteristic	All participants (*n* = 3201)	Participants without excessive sleepiness^*^ (*n* = 2402)
Apnea-hypopnea index – per 10 events/hour	1.15 (1.04, 1.26)	1.17 (1.02 1.33)
	*P* < 0.01	*P* = 0.02
Usual sleep duration – per hour less sleep	1.13 (1.01, 1.28)	1.22 (1.05, 1.43)
	*P* = 0.04	*P* < 0.01
Excessive daytime sleepiness (Epworth Sleepiness Scale score ≥ 11)	1.54 (1.15, 2.07)	–
	*P* < 0.01	

Values are odds ratio (95% confidence interval), adjusted for age, sex, and miles driven per year

^*^Absence of excessive sleepiness defined as Epworth Sleepiness Scale Score ≤ 10

For every hour decrease in usual sleep duration, the aOR for any motor vehicle crash increased by 13% in the overall population and by 22% in participants who did not report excessive sleepiness (Table 2). Crash risk was similar in those who reported sleeping 7 or 8 hours per night. Compared to those sleeping 7 or 8 hours per night (*n* = 2105), those who reported sleeping only 6 hours per night (*n* = 626) had a 33% higher aOR for any motor vehicle crash, while those sleeping 5 or fewer hours per night (*n* = 235) had a 47% higher aOR. Those who reported sleeping 9 or more hours per night (*n* = 235) had the lowest crash risk, with an aOR 24% lower than those sleeping 7 or 8 hours per night. Considering usual sleep duration of 7 to 8 hours per night as normative, the population-attributable fraction of motor vehicle crashes related to sleep duration of 6 or fewer hours per night was 9%.

**Fig. 3:**
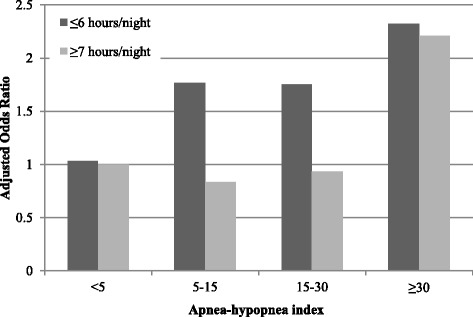
The joint association of sleep apnea and reported usual sleep duration to motor vehicle crash risk. The referent group is individuals with an apnea-hypopnea index < 5 and a usual sleep duration of ≥7 hours per night

Additional logistic regression models were performed to explore a possible interaction between sleep apnea and sleep duration on risk of motor vehicle crash. While it appears that increased crash risk in those with mild to moderate sleep apnea is present only in those sleeping 6 or fewer hours per night, and that increased crash risk in those with severe sleep apnea is present irrespective of usual sleep duration (Fig. 3), there was no significant interaction between sleep duration and sleep apnea severity with respect to crash risk (interaction term *p* = 0.65).

The estimated effects of sleep apnea or sleep duration on crash risk were not significantly different between men and women, and were not meaningfully affected by additional adjustment for body mass index, sleeping pill use, smoking history, usual alcohol consumption, or consumption of caffeinated beverages. Effects were also similar between younger and older participants, whether stratified on age 65 or age 70. Treatment for sleep apnea with continuous positive airway pressure, surgery, or an oral appliance at any time since the baseline sleep study was reported by 2.2% of the study sample, including 4.7% of those with AHI > 5, although treatment adherence and the timing of treatment initiation are not known. However, excluding from the analysis those who reported sleep apnea treatment had no meaningful impact on the association of either sleep apnea or sleep duration with crash risk. A sensitivity analysis that included the 1431 participants who completed the driving questionnaire but were excluded from analysis due to missing data for miles driven per year, adjusted for age and sex but not for miles driven, showed little change in effect estimates or tests of significance.

Self-reported excessive sleepiness was strongly associated with crash risk (Table 2 and Fig. 2). Despite this, adjustment for Epworth Sleepiness Scale score had little impact on the estimated risk associated with sleep apnea (aOR 1.13 per 10-unit increase in AHI) or usual sleep duration (aOR 1.12 per hour shorter sleep duration). Moreover, when the analysis is limited to the 75% of individuals without self-reported excessive sleepiness, the association of sleep apnea and usual sleep duration with motor vehicle crash risk was not diminished (Table 2). Indeed, the impact of short usual sleep duration on crash risk is most evident in those individuals who do not perceive themselves to be excessively sleepy.

## Discussion

In this community-dwelling cohort of middle-aged and older adults, the presence of sleep deficiency, whether due to short sleep duration or to sleep fragmentation from sleep apnea, was associated with a significantly increased risk of motor vehicle crashes, even among those who did not report excessive sleepiness. While one must be cautious in drawing a causal inference from observational data, the 19% estimate of motor vehicle crashes attributable to sleep deficiency in this analysis is consistent with estimates from the Virginia Tech 100 car naturalistic driving study that 22% of actual and near-crash incidents are attributable to drowsy driving [30], the Institute of Medicine’s estimate that 20% of serious motor vehicle crash injuries are attributable to sleep disorders and sleep deprivation [4], and the recent estimate that 21% of fatal crash injuries are attributable to drowsy driving [31].

The effect noted in the present study for those with severe sleep apnea is similar to that reported in clinically diagnosed sleep apnea patients [18, 19], with considerably smaller effects noted in those with milder sleep apnea. Individuals with mild to moderate sleep apnea and self-reported daytime sleepiness more closely resemble clinically diagnosed sleep apnea patients, however, and in this group the elevation in crash risk was also similar to that reported in clinically diagnosed patients. Short sleep duration may also increase crash risk among individuals with mild to moderate sleep apnea (Fig. 3), although further study with a larger sample is needed to verify this suggestive finding. The effects noted in this study are similar to those reported from the Wisconsin Sleep Cohort for risk of any motor vehicle crash over 5 years, although smaller than the risk observed in that cohort for multiple crashes [32]. There were too few participants reporting multiple crashes over the 1-year reporting interval for this study to replicate the Wisconsin analysis. Sleepiness is a widely recognized cause of motor vehicle crashes, a finding also confirmed in the present study. While sleep apnea is an important cause of excessive sleepiness, many individuals with sleep apnea in the general community do not report this symptom [13, 22]. In the present study, severe sleep apnea was associated with increased crash risk even among those without self-reported excessive sleepiness. Together with evidence that excess mortality in sleep apnea is independent of excessive sleepiness [33], these data indicate that treatment is warranted for many apparently asymptomatic patients who deny excessive sleepiness. These data support the conclusion that it is inappropriate to insist that the diagnosis and treatment of obstructive sleep apnea syndrome require excessive daytime sleepiness, which was standard practice for many years [34]. Such a requirement minimizes the prevalence of obstructive sleep apnea and would prevent the nearly half of patients with severe obstructive sleep apnea who do not report excessive sleepiness from obtaining an adequate diagnosis and thorough treatment, thereby perpetuating serious health problems and increased motor vehicle crash risk. These data support recent American Academy of Sleep Medicine diagnostic criteria that do not require the presence of excessive sleepiness for the diagnosis of severe obstructive sleep apnea [20].

In response to accumulating evidence of the potential adverse cardiovascular, metabolic, and performance consequences of insufficient sleep, the National Sleep Foundation, followed by the American Academy of Sleep Medicine and the Sleep Research Society, recently issued evidence-based recommendations that adults should sleep 7 or more hours per night for optimal health [35]. In the present study, habitual sleep durations of less than 7 hours per night are strongly associated with increased motor vehicle crash risk, even among those who do not report excessive sleepiness. A prior case–control study in a general population sample found that drivers in crashes involving death or hospitalization were more likely than control drivers to report having slept 5 or fewer hours the previous night, although the impact of habitual sleep duration was not assessed [36]. While reported sleepiness at the time of the crash was strongly associated with crash risk, usual sleepiness as measured by the Epworth Sleepiness Scale was not [36]. In a prospective study of new drivers aged 17–24 years, those who reported habitually sleeping 6 or fewer hours per night had an increased crash risk compared to those sleeping more than 6 hours per night, especially for crashes between the hours of 8 pm and 6 am. That study did not evaluate the role of sleepiness [37]. Like the present study, these prior studies suggest increased crash risk in very short sleepers. However, we further found that individuals who habitually sleep 9 or more hours per night have an even lower crash risk than those sleeping the recommended minimum of 7 hours per night, consistent with evidence that individuals who habitually sleep 7 to 8 hours per night carry greater sleep debt than those who habitually sleep 9 hours or more [38]. In commercial drivers, those with an actigraphy-measured sleep duration of 7 to 8 hours per night had a greater sleep propensity and poorer performance on sustained vigilance tasks compared to drivers whose usual sleep duration was greater than 8 hours per night [39]. Thus, the true fraction of motor vehicle crashes attributable to sleep restriction may well exceed 9%. In the present study, when those sleeping 9 or more hours per night are considered the referent group for crash risk, the estimated population-attributable fraction of crashes due to insufficient sleep rises to 30%. Together with the population-attributable fraction of motor vehicle crashes due to sleep apnea of 10%, this would raise the population-attributable fraction of motor vehicle crashes due to sleep deficiency to 40%, which is considerably higher than prior estimates based largely on self-reporting.

It remains to be determined why many people with severe sleep apnea or short sleep duration in this study did not report excessive sleepiness, even though their elevated crash risk suggests that their driving performance was impaired. Sleep deficiency might impair driving performance by decreasing vigilance even in the absence of increased sleepiness as measured by the Epworth Sleepiness Scale. Alternatively, usual sleepiness may be masked by the use of stimulants such as caffeine or by sympathetic activation secondary to sleep apnea or sleep deprivation. A reduced perception of sleepiness, particularly among those inured to the daytime consequences of chronic sleep deficiency, is also likely, and is consistent with the finding that subjective measures of sleepiness plateau after several days of restricted sleep duration, despite continued declines in objective measures of performance such as psychomotor vigilance [10, 11]. The Epworth Sleepiness Scale, although a clinical standard for two decades, may not be a sufficiently sensitive tool to detect a moderate degree of excessive sleepiness. Participants may also be reluctant to admit to excessive sleepiness, a symptom that many regard as pejorative, even in a research setting in which issues of liability are limited. This is likely to be even more problematic in settings in which an acknowledgment of sleepiness may have adverse occupational or legal consequences, e.g., with respect to maintaining a commercial driver’s license [40]. Objective tests of sleepiness may be more appropriate in this setting [41].

One potential limitation of this study is the use of self-report measures of driving and crash history. While motor vehicle crashes documented in police reports are the gold standard for accident reporting, past studies have shown that self-reporting is fairly accurate when reporting on accidents occurring within the prior year, as was done in this study. In a study of adult drivers aged 70 and older, there was substantial agreement between self-report and state-recorded motor vehicle crashes (kappa = 0.64) [42], with agreement as high as 85% between self- and police-reported crashes among younger drivers [43]. Moreover, the rate of motor vehicle crashes reported by the participants in this study (694 per 10,000 person-years or 696 per million miles driven) is quite similar to the rate of motor vehicle crashes reported by the AAA Foundation for Traffic Safety for U.S. adults (650 per 10,000 person-years or 578 per million miles driven) [44]. The strong association between crash risk and miles driven is also internally consistent. An important strength of this study is the prospective assessments of sleep apnea, sleep habits, and sleepiness, which are, therefore, not biased by subsequent crash history. Although these measures were made 1 year prior to the start of the reporting interval for driving history, they are fairly stable over this interval of time [27, 45] and variation would in any case bias toward a null result. Another important strength of the study is that it reflects the experience of a large community-dwelling cohort, rather than a cohort of clinically referred patients, and its results are, therefore, more generalizable to the public at large.

## Conclusions

Sleep deficiency resulting from either short sleep duration or sleep apnea is associated with a substantial increase in risk of motor vehicle crashes in the general population. These findings underscore the importance of developing accessible, valid, and objective biomarkers for determining impairment due to sleep deficiency. They further highlight the importance of ensuring that sleep apnea is effectively identified, diagnosed, and appropriately treated. Finally, in conjunction with the adverse metabolic consequences of insufficient sleep, these findings argue for public health efforts to improve adult sleep health.

## Abbreviations

AHI: Apnea-hypopnea index aOR Adjusted odds ratio
MVC: Motor vehicle crash
